# Analysis of mitochondrial alterations in Brazilian patients with sensorineural hearing loss using MALDI-TOF mass spectrometry

**DOI:** 10.1186/s12881-016-0303-5

**Published:** 2016-05-26

**Authors:** Rogério Marins Alves, Sueli Matilde da Silva Costa, Paulo Mauricio do Amôr Divino Miranda, Priscila Zonzini Ramos, Thiago Gibbin Marconi, Gisele Santos Oliveira, Arthur Menino Castilho, Edi Lúcia Sartorato

**Affiliations:** Center for Molecular and Genetic Engineering (CBMEG), University of Campinas (UNICAMP), Cidade Universitária Zeferino Vaz, Avenida Cândido Rondon 400, PO Box 6010, 13083-875 Campinas, São Paulo Brazil; Otology, Audiology and Implantable Ear Prostheses, University of Campinas (UNICAMP), Cidade Universitária Zeferino Vaz, Campinas, São Paulo Brazil

**Keywords:** mt-DNA mutations, Hearing loss, Molecular diagnosis

## Abstract

**Background:**

Mutations in the mitochondrial DNA (mtDNA) have been associated with aminoglycoside-induced and nonsyndromic deafness in different populations. In the present study, we investigated the contribution of mutations in mitochondrial genes to the etiology of hearing loss in a Brazilian sample.

**Methods:**

Using mass spectrometry genotyping technology, combined with direct sequencing, 50 alterations previously described in 14 mitochondrial genes were screened in 152 patients with sensorineural hearing loss and in104 normal hearing controls.

**Results:**

Fifteen known mitochondrial alterations were detected (G709A, A735G, A827G, G988A, A1555G, T4363C, T5628C, T5655C, G5821A, C7462T, G8363A, T10454C, G12236A, T1291C, G15927A). Pathogenic mutations in *MT-RNR1* and *MT-TK* genes were detected in 3 % (5/152) of the patients with hearing loss.

**Conclusions:**

This study contributed to show the spectrum of mitochondrial variants in Brazilian patients with hearing loss. Frequency of A1555G was relatively high (2.6 %), indicating that this mutation is an important cause of hearing loss in our population. This work reports for the first time the investigation and the detection of the tRNA^Lys^ G8363A mutation in Brazilian patients with maternally inherited sensorineural hearing loss.

## Background

Hearing loss (HL) is the most common sensory disorder, affecting one child in every 500 to 1000 at birth [1]. Genetic causes are estimated to represent 50–70 % of congenital deafness cases. Although most of hereditary HL cases are caused by mutations in nuclear genes, alterations in mitochondrial genes have been found in at least 5 % of patients with post-lingual nonsyndromic hearing loss [2].

Mutations in the mitochondrial DNA (mtDNA) can have structural and functional effects, including changes in RNA structure, reductions in the levels of mRNA or tRNA, and modification of tRNA. The deleterious effect of mtDNA mutations on energy metabolism appears to be the central pathogenic factor, although other important aspects of mitochondrial function may also play a role in disease pathogenesis, such as the generation of reactive oxygen species (ROS) and the regulation of programmed cell death, or apoptosis [3].

Many mitochondrial mutations have been associated with hearing loss, but the full range of phenotypic expression has not been well characterized, mainly due to the lack of consensus on audiological criteria and the low frequency of some mutations present in only a small number of cases. Further studies are necessary to establish the importance of the detected mutations for the diagnosis of hearing loss.

The most frequent mitochondrial mutations known to cause HL are the A1555G and C1494T alterations, which have been associated with aminoglycoside-induced and nonsyndromic deafness in families from many different ethnic backgrounds [4, 5]. Many other sequence variants have been proposed to be associated with hearing loss; however, most of them are not definitively related to hearing loss, because they have been found in normal hearing individuals and/or because of their mtDNA phylogeny [6].

At present, little is known about the incidence of mtDNA mutations in Brazilian patients. Previously published data have mainly focused on the A1555G mutation [7, 8] and there are no studies including a large number of deafness-associated mtDNA mutations.

In the present work, we investigated 50 alterations in 14 mitochondrial genes in order to estimate the frequency and involvement of known and putative mutations in Brazilian patients with sensorineural hearing loss. We also investigated the potential of the single nucleotide polymorphism (SNP) genotyping technology called iPLEX Gold MALDI-TOF MS (matrix-assisted laser desorption/ionization - time of fight mass spectrometry), developed by Sequenom Inc. (San Diego, CA), for the simultaneous analysis of mitochondrial alterations. This is the first study to undertake a systematic screening of Brazilian patients for mtDNA variations using MALDI-TOF MS.

## Methods

### Casuistics

We evaluated 152 unrelated patients of both genders (82 females and 70 males) with moderate to profound sensorineural HL. Samples from these individuals had been referred to the Human Molecular Genetics Laboratory of the Center for Molecular Biology and Molecular Engineering (CBMEG) at the University of Campinas (UNICAMP) by different organizations, in order to determine the etiology of HL. This work was a retrospective study using collected DNA samples from 2000 to 2013. Most of the patients included in the casuistic of this study were adults or young adults with progressive hearing loss with an age at diagnosis varying from childhood to early adulthood. Information of family history was scarcely available and not systematically for all patients.

In addition, 104 samples from normal hearing individuals were used as controls. The study was approved by the Research Ethics Committee of the Faculty of Medical Sciences of UNICAMP (Number 396/2006), and informed consent was obtained from all participants.

### DNA extraction

Genomic DNA was obtained from peripheral blood leukocytes using standard phenol-chloroform extraction. The molecular analysis was performed at CBMEG (UNICAMP). The purities and concentrations of the samples were determined using a NanoDrop® 8000 spectrophotometer (Thermo Scientific) and a Qubit® 2.0 fluorometer (Invitrogen), respectively. All the samples showed A260/280 ratios between 1.8 and 2.0. Genomic DNA samples were diluted to obtain a final concentration of 5 ng/μL.

### Mutation analysis of the DFNB1 locus

Mutations in the DFNB1 locus, involving the *GJB2* and *GJB6* genes, are the most common cause of autosomal recessive nonsyndromic hearing loss (ARNSHL). All the samples were therefore previously tested for the presence of mutations in the coding region of *GJB2* and for the intronic mutation IVS1 + 1G > A, using PCR, followed by direct sequencing using ABI BigDye Terminator and analysis with an ABI PRISM® 3700 DNA sequencer (Applied Biosystems) [9]. A multiplex PCR methodology was used to detect del(GJB6-D13S1830) and del(GJB6-D13S1854), according to the procedures reported previously [10, 11]. Patients with HL due to mutations in the DFNB1 locus were ruled out for subsequent analyses.

### Mutation analysis of the mtDNA

A total of 50 mitochondrial alterations related to nonsyndromic HL, described in the Human Mitochondrial Genome Database (MITOMAP) [12], were screened using the iPLEX Gold/MALDI-TOF MS technology (Sequenom Inc., San Diego, CA). These alterations are spread in a total of 14 genes as listed in Table 1.

**Table 1:** mtDNA alterations analyzed by MALDI-TOF mass spectrometry

Gene	Alteration	RNA
*MT-TF*	A622G	tRNA^Phe^
	A636G	tRNA^Phe^
	T642C	tRNA^Phe^
*MT-RNR1*	T669C	12S rRNA
	G709A	12S rRNA
	A735G	12S rRNA
	A745G	12S rRNA
	A792G	12S rRNA
	A801G	12S rRNA
	A827G	12S rRNA
	A839G	12S rRNA
	A856G	12S rRNA
	G988A	12S rRNA
	T990C	12S rRNA
	T1005C	12S rRNA
	A1027G	12S rRNA
	A1116G	12S rRNA
	T1180G	12S rRNA
	C1192A	12S rRNA
	C1226G	12S rRNA
	T1291C	12S rRNA
	C1310T	12S rRNA
	A1331G	12S rRNA
	A1374G	12S rRNA
	T1452C	12S rRNA
	A1453G	12S rRNA
	C1494T	12S rRNA
	C1537T	12S rRNA
	A1555G	12S rRNA
*MT-TI*	A4316G	tRNA^Ile^
*MT-TQ*	T4336C	tRNA^Gln^
	T4363C	tRNA^Gln^
*MT-TW*	G5540A	tRNA^Trp^
	A5568G	tRNA^Trp^
*MT-TA*	T5587C	tRNA^Ala^
	T5628C	tRNA^Ala^
	T5655C	tRNA^Ala^
*MT-TC*	T5802C	tRNA^Cys^
	G5821A	tRNA^Cys^
*MT-TS1*	A7445C	tRNA^Ser (UCN)^
	A7456G	tRNA^Ser (UCN)^
	C7462T	tRNA^Ser (UCN)^
*MT-TK*	G8363A	tRNA^Lys^
*MT-TR*	T10454C	tRNA^Arg^
*MT-TH*	G12183A	tRNA^His^
*MT-TS2*	C12224T	tRNA^Ser (AGY)^
	G12236A	tRNA^Ser (AGY)^
*MT-TE*	A14693G	tRNA^Glu^
*MT-TT*	T15908C	tRNA^Thr^
	G15927A	tRNA^Thr^

All the results were validated by direct sequencing, using the same primers used in iPLEX Gold locus-specific PCR reactions. DNA sequencing was performed with the ABI BigDye™ Terminator v3.1 Cycle Sequencing Kit on an ABI PRISM® 3700 DNA Analyzer (Applied Biosystem), according to manufacturer’s instructions. The Chromas Lite v2.0 (Technelysium Pty Ltd) and CLC Sequence Viewer v6 (CLC bio) free softwares were used to analyze sequencing data.

### iPLEX Gold/MALDI-TOF MS

Based on the sequence data available from MITOMAP [12], MassARRAY Assay Designer software (Sequenom Inc., San Diego, USA) was used to design PCR and single base extension (SBE) primers for all 50 mtDNA variants investigated. The iPLEX Gold technology consists of an initial locus-specific PCR reaction, followed by SBE using mass-modified dideoxynucleotide terminators of an oligonucleotide primer that anneals immediately upstream of the polymorphic site of interest. Matrix-assisted laser desorption/ionization time-of-flight mass spectrometry (MALDI-TOF MS) is used to identify the corresponding allele according to the mass of the extended primer.

The optimal amplicon size was set to 80–120 bp. A 10-mer tag (5’-ACGTTGGATG-3’) was added to the 5’ end of each PCR primer to avoid confusion in the mass spectrum, and SBE primers were 5’ tailed with nonhomologous sequences of varying lengths to create large enough mass differences between the different SBE products to be detected by MALDI-TOF MS. In order to avoid interaction among the primers of the 83-plex (83 reactions), the MassARRAY Assay Designer software divided the PCR amplification and the SBE into 5 multiplex reactions.

The whole process was performed according to the manufacturer’s instructions for the multiplex reactions, including the PCR amplification, the shrimp alkaline phosphatase (SAP) treatment, and the primer extension reactions using the iPLEX Gold assay. The reaction products were then dispensed onto a 384-SpectroCHIP using the MassARRAY Nanodispenser, and were analyzed on the MassARRAY platform. Mass signals for the different alleles were captured with high accuracy by MALDI-TOF MS. Typer software v4.0 (Sequenom Inc., San Diego, CA) was used to process the raw data obtained from the assays.

## Results

The iPLEXGold/MALDI-TOF MS method was used to screen 50 mtDNA mutations associated with hearing loss. A total of 256 samples obtained from 152 patients with sensorineural HL and 104 normal hearing controls were analyzed. Fifteen different mtDNA alterations were detected: G709A, A735G, A827G, A988G, A1555G, T4363C, T5628C, T5655C, G5821A, C7462T, G8363A, T10454C, G12236A, T1291C, G15927A (Table 2). Based on the MITOMAP database, status was considered ‘Confirmed’ if at least two or more independent studies have reported the pathogenicity of a specific mutation. Ambiguous substitutions were categorized as ‘Unclear’ or ‘Conflicting’. ‘Reported’ status indicates that one or more reports have considered the mutation as possibly pathogenic (Table 2). Only 3 % (5/152) of the patients had ‘Confirmed’ mutations (4/A1555G and 1/G8363A). Two alterations (C7462T and A735G) were only found in the control group, seven only in patients with HL and six were found in both patients and control group. The T4363C alteration was detected in all the individuals analyzed (Table 3). All the results were confirmed by direct sequencing and showed a 100 % match for it.

**Table 2:** Summary of alterations identified only in patients with sensorineural HL

Patient	Alteration	Gene	Haplogroup	Gender	Hetero/Homoplasmy	Status^a^
Mit05	A827G	*MT-RNR1*	L3 – R	M	Homo	Conflicting
Mit12	A827G	*MT-RNR1*	L3 – R	M	Homo	Conflicting
Mit13	A827G	*MT-RNR1*	L3 - R	F	Homo	Conflicting
Mit15	**G8363A** - G15927A	*MT-TK* *MT-TT*	L3 - X - J	M	Homo	**Confirmed** Possible helper
Mit17	**A1555G**	*MT-RNR1*	L3 - D	M	Homo	**Confirmed**
Mit18	**A1555G**	*MT-RNR1*	L3 - D	M	Homo	**Confirmed**
Mit19	A827G	*MT-RNR1*	L3 - R	M	Homo	Conflicting
Mit21	A827G	*MT-RNR1*	L3 - R	F	Homo	Conflicting
Mit24	G709A	*MT-RNR1*	L3 - R - T	F	Homo	Reported
Mit25	T5655C	*MT-TA*	L1/L2	M	Hetero	Reported
Mit27	A827G	*MT-RNR1*	L3	M	Homo	Conflicting
Mit29	A827G	*MT-RNR1*	L3 - R	M	Homo	Conflicting
Mit32	A827G	*MT-RNR1*	L3 - R	F	Homo	Conflicting
Mit33	**A1555G**	*MT-RNR1*	L1/L2	M	Homo	**Confirmed**
Mit34	G709A	*MT-RNR1*	L3 - R - T	F	Homo	Reported
Mit36	G709A	*MT-RNR1*	L3 - R - T	F	Hetero	Reported
Mit43	G709A	*MT-RNR1*	L1/L2	M	Hetero	Reported
Mit47	**A1555G**	*MT-RNR1*	L3 - R - H	F	Homo	**Confirmed**
Mit48	**A1555G**	*MT-RNR1*	L1/L2	M	Homo	**Confirmed**
Mit49	A827G	*MT-RNR1*	L3 - R	M	Homo	Conflicting
Mit54	G709A	*MT-RNR1*	L3 - R - T	M	Hetero	Reported
Mit57	A827G	*MT-RNR1*	L3 - R	M	Homo	Conflicting
Mit59	A827G	*MT-RNR1*	L3 - R	F	Homo	Conflicting
Mit63	G5821A	*MT-TC*	L1/L2	M	Homo	Reported
Mit74	A827G	*MT-RNR1*	L3 - R - T	F	Homo	Conflicting
Mit77	G12236A	*MT-TS2*	L1/L2	F	Homo	Reported
Mit85	G709A	*MT-RNR1*	L3 - R	M	Homo	Reported
Mit86	A827G	*MT-RNR1*	L3	M	Homo	Conflicting
Mit91	G709A	*MT-RNR1*	L3	F	Homo	Reported
Mit94	A827G	*MT-RNR1*	L3 - R	F	Homo	Conflicting
Mit96	G709A	*MT-RNR1*	L3 - R	M	Homo	Reported
Mit97	A827G	*MT-RNR1*	L3 - R	M	Homo	Conflicting
Mit98	T5655C	*MT-TA*	L1/L2	F	Homo	Reported
Mit99	A827G	*MT-RNR1*	L3 - R	M	Homo	Conflicting
Mit100	T5655C	*MT-TA*	L3	F	Hetero	Reported
Mit102	G709A	*MT-RNR1*	L3 - R - T	F	Homo	Reported
Mit108	T5628C-G988A	*MT-TA* *MT-RNR1*	L3	F	Homo	Reported Reported
Mit117	G709A	*MT-RNR1*	L3	M	Homo	Reported
Mit118	T1291C	*MT-RNR1*	L1/L2	F	Homo	Unclear
Mit129	A827G	*MT-RNR1*	R - T	M	Homo	Conflicting
Mit130	G709A	*MT-RNR1*	L3 - R	F	Hetero	Reported
Mit133	T10454C	*MT-TR*	L1/L2	M	Homo	Reported
Mit135	T5655C	*MT-TA*	L1/L2	M	Homo	Reported
Mit137	A827G	*MT-RNR1*	L3 - R	F	Homo	Conflicting
Mit140	G709A	*MT-RNR1*	L3	M	Homo	Reported
Mit144	A827G	*MT-RNR1*	L3 - R	M	Homo	Conflicting
Mit145	A827G	*MT-RNR1*	L3 - R	M	Homo	Conflicting
Mit149	G709A	*MT-RNR1*	L3	M	Homo	Reported
Mit151	A827G	*MT-RNR1*	L3 – T	M	Homo	Reported

^a^Based on the MITOMAP database; ‘Reported’ status indicates that one or more reports have considered the mutation as possibly pathologic. ‘Confirmed’ (bold) indicates that two or more independent studies had reported about the pathogenicity of specific mutation

**Table 3:** Frequency of mitochondrial DNA alterations identified in patients with sensorineural HL and normal hearing individuals

Alteration	Patients	Normal controls
A1555G.	4/152 (2.6 %)	0
A827G.	20/152 (13.2 %)	9/104 (8.6 %)
G709A.	14/152 (9.2 %)	13/104 (12.5 %)
G8363A.	1/152 (0.7 %)	0
G15927A.	1/152 (0.7 %)	0
G12236A.	1/152 (0.7 %)	0
T10454C.	1/152 (0.7 %)	0
T5628C.	1/152 (0.7 %)	0
A988G.	1/152 (0.7 %)	0
G5821A.	1/152 (0.7 %)	2/104 (1.9 %)
T5655C	4/152 (2.6 %)	4/104 (3.8 %)
T1291C	1/152 (0,7 %)	6/104 (5,8 %)
C7462T	0	1/104 (1 %)
A735G	0	1/104 (1 %)
T4363C	152 (100 %)	104 (100 %)

## Discussion

Fifteen known mitochondrial mutations were detected in this study. Seven of them, A988G, A1555G, T5628C, G8363A, T10454C, G12236A and G15927A, were found only in the patients with HL, supporting the pathogenicity of these mutations. The A1555G (4/152) classified as ‘Confirmed’ by MITOMAP database was found in the patients with moderate to profound sensorineural HL. The A1555G mutation in the highly conserved decoding site of the mitochondrial 12S rRNA alters the secondary structure, so that it resembles the corresponding region of bacterial 16S rRNA, increasing the risk of hearing impairment due to aminoglycosides exposure [13]. Frequency of A1555G was relatively high (2.6 %) indicating that this alteration should be included in the mutation group routinely investigated in Brazilian patients with sensorineural HL [14]. Aminoglycoside-induced and nonsyndromic deafness has been shown to have a genetic susceptibility and the pathogenic mitochondrial 12S rRNA A1555G mutation was identified as the primary factor underlying the hearing loss, particularly in countries where aminoglycoside antibiotics are commonly used even for minor infections [15]. However, the A1555G mutation has also been reported in hearing-impaired patients with no history of exposure to aminoglycoside antibiotics, suggesting that in some carriers of this mutation aminoglycoside exposure is not necessary for the occurrence of HL, and additional environmental and/or genetic risk factors may influence the penetrance and expressivity [16].

In the present study, none of the patients with HL carrying the A1555G mutation reported the use of aminoglycosides.

One patient (Mit15) carrying the G15927A mutation in the tRNA^Lys^ gene also presented the known pathogenic G8363A mutation in the tRNA^Lys^ gene, apparently in homoplasmy after MALDI-TOF MS and sequencing. The G8363A mutation has been reported for the first time in two unrelated families, causing encephalomyopathy, sensorineural hearing loss and hypertrophic cardiomyopathy [17]. Previous studies have shown that the same mutation can determine a large phenotypical variability ranging from MERRF-like syndrome, maternally inherited cardiomyopathy, deafness, autism, or Leigh syndrome [18–20]. The G8363A abolishes a highly conserved pairing in the aminoacyl stem of the tRNA^Lys^ altering the overall secondary structure of the molecule and consequently impairs the activity of peptidyl-tRNA hydrolase, which is critical for protein translation [21]. On the other hand, the G15927A mutation abolishes the conserved base pairing 28C-42G on the anticodon stem of tRNA^Thr^ and consequently alters the metabolism of this tRNA. The G15927A have been implicated in enhancing the penetrance and expressivity of the deafness-associated 12S rRNA A1555G mutation [22, 23]. However, the association of the G15927A mutation with coronary heart disease is controversial [24].

The patient Mit15 carrying G8363A and G15927A mutations was a Brazilian 26-year-old man who had bilateral progressive sensorineural HL starting at around 11 years of age, and subsequently underwent cochlear implantation at 22 years old. To date, no other clinical signs or symptoms were detected. Family history was remarkable for two brothers who presented similar phenotype and carrying the same mutations in homoplasmy. Curiously, the mother presented G15927A in homoplasmy but G8363A in heteroplasmy and she has normal audiological examinations. The G8363A mutation is being reported for the first time in a Brazilian patient with sensorineural HL. Nonetheless, more studies are necessary to determine the frequency of this mtDNA mutation in our country.

The alterations 12S rRNA G988A, tRNA^Ala^ T5628C, tRNA^Arg^ T10454C, tRNA^Ser(AGY)^ G12236A were detected in four patients with sensorineural HL. These mtDNA mutations can contribute to the phenotype because (1) they affect highly conserved nucleotides in mitochondrial genes (conservation index >50 %), (2) they were found in maternal inheritance (some were also found as sporadic cases), (3) None of these mutations were found in normal hearing controls and (4) they lead to structural alterations and affect the function of rRNA or may impair mitochondrial translation by reducing the availability of functional mt-tRNAs [25–28]. Nevertheless, among these mutations only G12236A have been considered deleterious [26, 29]. The G988A, T5628C and T10454C mutations were reported as a possible risk factor for deafness or modulating factors of the phenotypic expression of pathogenic mutations, indicating that these alterations themselves are not sufficient to cause clinical symptoms.

The A827G mutation was the most frequent in patients with HL, being present in 20 out of 152 (13 %). In the normal controls, the frequency was 9 out of 104 (8.6 %). A significant difference in the proportion of individuals with the A827G mutation among the patients with HL (13 %) and normal hearing controls (8.6 %) was observed. Similar results were found in other studies in different populations [30–32]. The A827G mutation has been implicated in aminoglycoside ototoxicity and nonsyndromic HL. Xing et al. (2006) [33] studied two unrelated Chinese families with HLs, and found that this mutation was present in all the maternal relatives, while not all mutation carriers were affected. The A827G mutation is located at the highly conserved A-site of the 12S rRNA gene. It is possible that the alteration of the tertiary or quaternary structures of this rRNA by the A827G mutation may lead to mitochondrial dysfunction, thereby playing a role in the pathogenesis of hearing loss and aminoglycoside hypersensitivity. However, the incomplete penetrance indicates that the A827G mutation alone is not sufficient to produce clinical phenotype, requiring the involvement of modifying factors including aminoglycosides, mitochondrial haplotypes or nuclear modifier genes [34–36]. On the other hand, the contribution of this variant to HL remains controversial, given its high frequency in the normal population [32].

The two most important mitochondrial genes associated with nonsyndromic HL are the *MT-RNR1*and *MT-TS1*. However, genotyping of mutations using mass spectrometry resulted in discernment of alterations in six other genes (*MT-TK*, *MT-TT*, *MT-TA*, *MT-TS2*, *MT-TC*, and *MT-TR*). The iPLEX Gold/MALDI-TOF MS methodology enables automated large-scale mutation screening, providing accuracy and high-throughput. The data are easy to interpret, due to the use of a well-established protocol, although the results should be confirmed by other methods. The main drawbacks of the iPLEX assay are the requirement for specific equipment and the cost of the MassARRAY instrumentation. Nonetheless, the technique offers a high level of flexibility and a low cost per genotype. The use of MALDI-TOF MS could be especially appealing for laboratories with medium to high-throughput activities.

Due to great genetic heterogeneity, the molecular diagnosis of different types of hereditary hearing loss has been challenging. The spectrum of molecular tests available is growing significantly but it is also critical to note that they are not available for all deafness genes. Therefore, it is important to know the most frequent mutations in the population of interest to the elaboration of specific high-throughput panels containing only the commonest mutations, in order to reduce costs and allow the rapid screening of large number of patients in routine tests. MtDNA analysis can contribute to the diagnosis of syndromic HL before the onset of clinical symptoms; can distinguish individuals with mitochondrial mutations who are at risk for HL when treated with aminoglycosides; and allow for more accurate genetic counseling.

## Conclusion

Systematic analysis of mtDNA mutations by iPLEX Gold/MALDI-TOF MS methodology in Brazilian patients with sensorineural HL contributed for provide the range of main alterations of mitochondrial genes in Brazilian patients with HL. Fifteen known mitochondrial mutations were detected in this study. At least two of them (A1555G and G8363A) should be included in the screening of genetic alterations related to HL. The rare G8363A mutation in mtDNA tRNA^Lys^ gene was reported for the first time in a Brazilian patient with sensorineural HL.
